# Developmental trajectory of contextual learning and 24-h acetylcholine release in the hippocampus

**DOI:** 10.1038/srep03738

**Published:** 2014-01-17

**Authors:** Kenkichi Takase, Yuya Sakimoto, Fukuko Kimura, Dai Mitsushima

**Affiliations:** 1 Department of Physiology, Yokohama City University Graduate School of Medicine, 3-9 Fukuura Kanazawa-ku, Yokohama 236-0004, Japan; 2 Department of Anatomy, Toho University School of Medicine, 5-21-16 Omori-Nishi Ota-ku, Tokyo 143-8540, Japan; 3 Department of Systems Neuroscience, Yamaguchi University Graduate School of Medicine, 1-1-1 Minami-Kogushi, Ube 755-8505, Japan

## Abstract

To determine the developmental trajectory of hippocampal function in rats, we examined 24-h changes in extracellular acetylcholine (ACh) levels and contextual learning performance. Extracellular ACh significantly correlated with spontaneous behavior, exhibiting a 24-h rhythm in juvenile (4-week-old), pubertal (6-week-old), and adult (9- to 12-week-old) rats. Although juveniles of both sexes exhibited low ACh levels, adult males had higher ACh levels than adult females. Moreover, juveniles exhibited much more spontaneous activity than adults when they showed equivalent ACh levels. Similarly, juveniles of both sexes exhibited relatively low contextual learning performance. Because contextual learning performance was significantly increased only in males, adult males exhibited better performance than adult females. We also observed a developmental relationship between contextual learning and ACh levels. Scopolamine pretreatment blocked contextual learning and interrupted the correlation. Since long-term scopolamine treatment after weaning impaired contextual learning in juveniles, the cholinergic input may participate in the development of hippocampus.

Both spatial and contextual learning require acetylcholine (ACh) release in the hippocampus^1^,^2^. ACh release that increases during learning^3^,^4^ appears to orchestrate many hippocampal functions such as the generation of theta waves, induction of long-term potentiation (LTP) in CA1 synapses, and cell proliferation in the dentate gyrus^5^,^6^,^7^,^8^.

Several lines of evidence suggest sex-based differences in the hippocampal learning of various mammalian species, including humans^9^,^10^. Compared with females, males exhibit superior hippocampal learning in both contextual and spatial tasks, including mental rotation^11^,^12^,^13^,^14^,^15^,^16^,^17^,^18^. As a mechanism underlying the differences, we demonstrated ACh levels in the dorsal hippocampus: rats of both sexes exhibited behavior-linked circadian changes in extracellular ACh levels, and males exhibited higher ACh levels than females^19^,^20^. In addition, neonatal treatment with estrogen masculinized ACh levels and behavior, suggesting sexual differentiation of the septo-hippocampal cholinergic system^21^.

Although the developmental trajectory of sex-specific contextual learning is still unknown, spatial learning seems to associate with pubertal status. In maze learning, no differences between sexes were observed prior to puberty; however, males made fewer errors than females in post-puberty^22^. Moreover, the location-specific firing of hippocampal pyramidal cells that depend on ACh levels becomes adult-like at approximately 50 days of age, suggesting a developmental increase in ACh in male rats^23^,^24^. Immunocytochemical study further demonstrated a distinct increase in CA1 cholinergic innervation in peripubertal male rats^25^. Although this developmental change is not completely unknown in females, these results imply the maturation of ACh release and the linkage of ACh levels to behavior around puberty^17^,^20^,^21^. Here we report peripubertal changes in 24-h ACh levels and contextual learning in rats of both sexes, suggesting a developmental relationship between the ACh levels and contextual learning. Simultaneous monitoring of ACh levels with spontaneous locomotor activity further allowed us to analyze the development of ACh release profiles in both sexes.

## Results

### Extracellular acetylcholine levels

**Figure 1:**
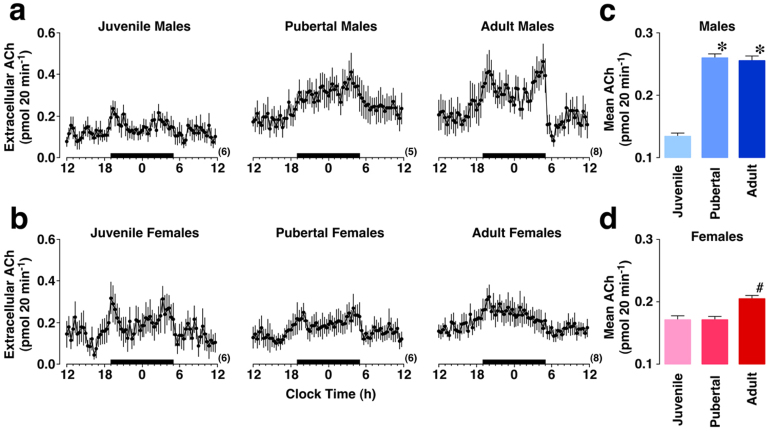
Postnatal changes in extracellular ACh in the dorsal hippocampus. Horizontal black bars indicate the dark phase. ACh levels in male (a) and female (b) rats clearly follow a 24-h rhythm. Overall ACh levels were statistically summarized in male (c) and female (d) groups. Parentheses indicate the number of rats. * *P* < 0.0001 vs. juvenile males. # *P* = 0.01 vs. juvenile females. Adult rats exhibited significant sex-specific differences (*P* < 0.0001). Data represent the means ± SEM.

Figure 1 shows 24-h profiles of extracellular ACh levels in six age groups. Three-way ANOVA revealed a significant main effect of daily change (*F*_(71, 2343)_ = 21.550, *P* < 0.0001), age × time interaction (*F*_(142, 2343)_ = 1.537, *P* < 0.0001), and sex × age × time interaction (*F*_(142, 2343)_ = 1.628, *P* < 0.0001). The difference between the sexes was analyzed using *post-hoc* ANOVA for each age. Although the sex difference was non-significant in juvenile (*P* = 0.42) and pubertal rats (*P* = 0.24), adult males exhibited significantly greater ACh levels than adult females (*P* < 0.0001).

The developmental change was also analyzed for each sex. Low ACh levels in juvenile males increased significantly in pubertal (*P* < 0.0001) and adult males (*P* < 0.0001). In contrast, low ACh levels in juvenile females did not increase in pubertal females (*P* = 0.99), but significantly increased in adult females (*P* = 0.0102). A significant time-dependent change was consistently observed in all age groups (*P* < 0.0001).

### Correlation between acetylcholine and locomotor activity

**Figure 2:**
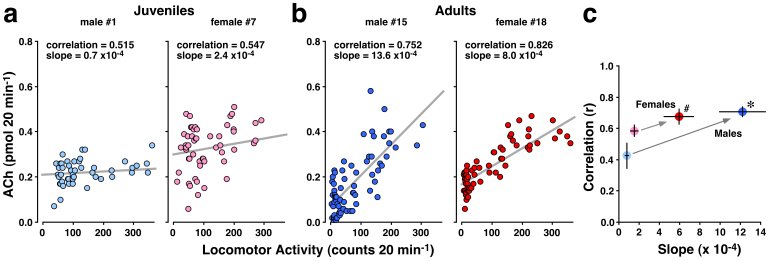
Correlation between ACh levels and spontaneous locomotor activity in juvenile and adult rats. Simple linear regression analysis showed that the data from 2 juvenile rats (a) had a gentle slope of the fit line, while the data from 2 adult rats (b) had a steep slope. (c) The slope of best-fit line was steeper for adult rats of both sexes than for juveniles. Adult males exhibited a significantly higher correlation coefficient than juvenile males and a steeper slope of the best-fit line than adult females (*P* < 0.01). *P < 0.01 compared with juvenile males (both correlation and slope). ^#^*P* < 0.01 compared with juvenile females (slope only). Data in (c) represent the means ± SEM.

Figure 2 depicts a positive correlation between the ACh levels and spontaneous locomotor activity in four representative cases of juvenile (n = 2) and adult (n = 2) rats. In simple linear regression analysis, the ACh levels in juveniles (#1 and #7) are weakly correlated with spontaneous locomotor activity (Fig. 2a), while the ACh levels in adult rats (#15 and #18) correlated strongly with the activity (Fig. 2b). The correlation coefficient appears to be higher and the slope of the fit line seems to be steeper in adult rats than in juvenile rats (Fig. 2c). In the correlation coefficient, two-way ANOVA revealed a significant main effect of development (*F*_(1, 18)_ = 11.789, *P* = 0.003); however, the main effect of sex (*F*_(1, 18)_ = 1.429, *P* = 0.25) and the interaction between sex and development (*F*_(1, 18)_ = 3.039, *P* = 0.10) were not significant. The slope of the best-fit line indicates that the main effects of development (*F*_(1, 18)_ = 40.984, *P* < 0.0001) and sex (*F*_(1, 18)_ = 5.003, *P* = 0.038), as well as the interaction between development and sex (*F*_(1, 18)_ = 8.055, *P* = 0.011) were significant. In males, both correlation and slope in juveniles were significantly improved in adults (*P* < 0.01). In females, moderate slope in juveniles improved in adults (*P* < 0.01), while the relatively high correlation in juveniles was unchanged in adults. Data from adult males had a significantly steeper slope than data from adult females (*P* < 0.01), indicating a sex-specific property of ACh release.

### Contextual fear learning

**Figure 3:**
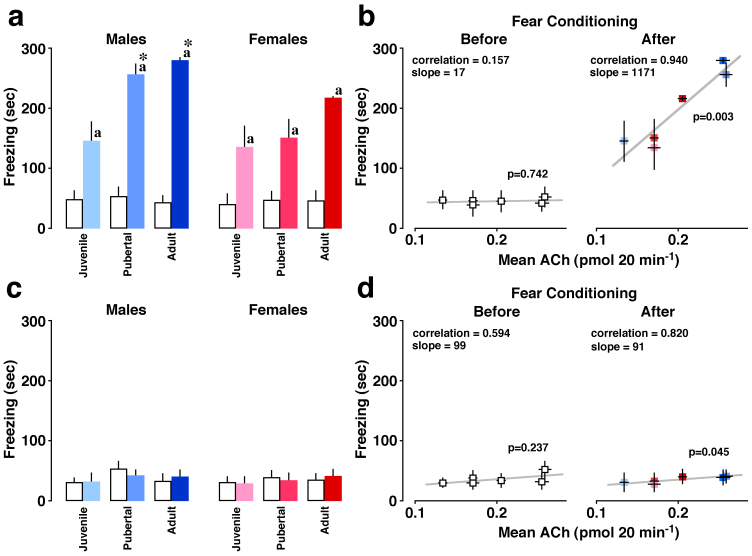
Developmental relationship between contextual learning and ACh levels. Open and filled bars represent before and after conditioning, respectively (n = 6 each). Blue and red color represents conditioned males and females, respectively. The total freezing time during the 5-min testing period is shown. (a) All groups of rats exhibited longer freezing times after conditioning (^a^ *P* < 0.05 *vs.* before). Pubertal and adult males exhibited longer freezing times than juveniles (*P < 0.01), while the change was not significant in females. Pubertal and adult males exhibited longer freezing times than corresponding females (*P* < 0.05 and 0.0001, respectively). (b) The freezing time was plotted versus mean ACh levels in 6 age groups. Conditioning increased correlation (*P* < 0.01) and induced steeper slope. (c) Scopolamine pretreatment consistently blocked contextual learning in all groups. (d) The freezing time in scopolamine-pretreated rats was plotted versus ACh level in six groups. Data represent the means ± SEM.

Figure 3 shows the total freezing time before and after fear conditioning. Three-way ANOVA revealed significant differences in sex (*F*_(1, 60)_ = 6.786, *P* = 0.012), age (*F*_(2, 60)_ = 6.793, *P* = 0.002), conditioning (*F*_(1, 60)_ = 154.651, *P* < 0.0001), sex × conditioning interaction (*F*_(1, 60)_ = 5.290, *P* = 0.025), and age × conditioning interaction (*F*_(2, 60)_ = 6.572, *P* < 0.003). The difference between the sexes was analyzed using *post-hoc* ANOVA for each age before and after conditioning.

The developmental change was also analyzed for each sex. After conditioning, sex differences were observed in pubertal (*P* < 0.05) and adult rats (*P* < 0.0001), but not in juveniles. Pubertal and adult males exhibited greater total freezing time than juvenile males (*P* < 0.01). In contrast, female rats did not exhibit a significant developmental change in total freezing time. Neither developmental changes nor sex differences were observed in rats before conditioning.

Freezing time was plotted with mean ACh levels in age groups (Fig. 3b). Although the correlation was low before conditioning, a significant correlation (*R* = 0.94, *P* = 0.0026) was observed after conditioning. We also observed a mathematical relation between freezing time and ACh levels after conditioning: a steeper slope was observed between the two parameters [Freezing time (sec) = 1171 ACh (pmol/20 min) + 36].

Pretreatment with scopolamine consistently impaired contextual learning in all groups, and severely attenuated the difference among groups (Fig. 3c). Three-way ANOVA revealed no significant main effects or interactions (*F*_(2, 60)_ = 0.860, *P* = 0.43). Freezing time in scopolamine-pretreated rats was plotted versus mean ACh levels in six age groups (Fig. 3d). Although both correlation and slope were low before conditioning, scopolamine-pretreated rats exhibited a weak correlation (*P* = 0.045) after conditioning.

**Figure 4:**
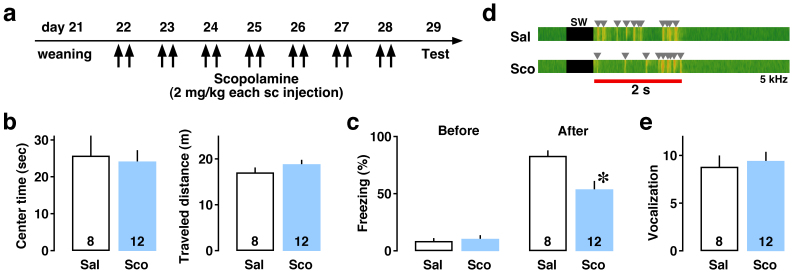
Long-term scopolamine treatment affects contextual learning in juvenile males. (a) Saline (Sal) or scopolamine (Sco) was subcutaneously injected twice daily (at 9:00 and 19:00) for a week. One day after the final injection, the rats were tested for behavioral tasks. (b) In open field test, the long-term Sco treatment did not affect center time or total traveled distance. (c) In fear conditioning test, Sco-treated juveniles exhibited lower freezing rate than Sal-treated controls after the conditioning (*P = 0.012). (d) Sound analysis extracted the number of vocalization during the period of 2-s foot shock (red bar). Examples of visualized vocalization (at 5 kHz) were shown. SW indicates switching period of shock device. (e) The number of vocalization was not significant between groups. The number of rats in each group is shown at the bottom of each bar. Error bars indicate ± SEM.

To further examine developmental role of ACh, scopolamine was injected twice daily for 7 consecutive days after weaning. One day after the final injection, the juvenile males were tested for behavioral tasks (Fig. 4a). In open field test (Fig. 4b), the long-term scopolamine treatment did not affect center time (*P* = 0.80) and spontaneous traveled distance (*P* = 0.12). In fear conditioning test (Fig. 4c–e), both groups of rats showed low freezing rate before the conditioning (*P* = 0.60). After the conditioning, however, the long-term scopolamine treatment decreased the freezing rate (Fig. 4c, *P* = 0.012). Sound analysis (Fig. 4d) further indicated that the treatment did not affect the number of vocalization at the conditioning (Fig. 4e, *P* = 0.71).

## Discussion

We examined the development of hippocampal ACh levels and contextual learning. Extracellular ACh levels in the dorsal hippocampus are low in juveniles, but increase significantly in adults. Simultaneous monitoring of ACh levels and spontaneous locomotor activity further demonstrated the development of ACh release. Although both juvenile and adult rats exhibited significant correlations between ACh levels and spontaneous activity, juveniles exhibited much more spontaneous activity than adults when they showed equivalent ACh levels. Similarly, low contextual learning performance in juveniles of both sexes significantly increased, especially in males, demonstrating a developmental trajectory of hippocampal function. We also observed a developmental relationship between contextual learning and ACh level.

Because ACh is known to induce LTP without a stimulatory electrode in hippocampal slice^5^, sexual dimorphism with regard to LTP may contribute to the sex difference in hippocampal functions. In fact, a robust sex-based difference exists in the magnitude of LTP induced in the hippocampal formation of anesthetized rats^26^,^27^, and multiple trains of tetani induce LTP in the hippocampus of male rats more effectively than in female rats^28^. Moreover, as a learning-dependent mechanism of plasticity, we found that contextual learning requires the synaptic delivery of AMPA receptors into CA1 synapses, and ACh triggers delivery through a muscarinic receptor-mediated mechanism^29^,^30^. Given that genetic ablation of newly formed neurons revealed that contextual learning requires neurogenesis in the dentate gyrus^31^, both AMPA receptor delivery into CA1 synapses and neurogenesis in the dentate gyrus may contribute to sex difference in hippocampal function^32^.

We monitored *in vivo* ACh levels in the CA1–CA3 subregion in the dorsal hippocampus, where primary neurons contribute to both spatial^33^ and contextual learning^29^,^34^. At the cellular level, the firing of CA1–CA3 pyramidal neurons correlates clearly with their location in space, contributing to form a cognitive map in the hippocampus^35^,^36^,^37^,^38^. The location-specific firing of hippocampal pyramidal cells was dose-dependently slowed and degraded by muscarinic blockade^24^. This firing becomes adult-like at approximately 50 days of age; spatial firing recorded in the CA1 layer of rats younger than 50 days of age is broad and diffuse, with a high rate of background firing. In comparison, background firing in rats older than 50 days of age is considerably lower— essentially zero in most cases^23^. The increased accuracy of location-specific firing paralleled a decrease in background firing^23^. Because spatial information is an element necessary to the formation of contextual memory, these results may suggest that increases in ACh levels improve both spatial and contextual learning by changing the location-specific firing of CA1 neurons.

Krasnoff and Weston^22^ reported sex differences in the maze performances of rats in relation to pubertal status; no sex differences were found prior to puberty, but males made fewer maze errors than females after puberty. Consistently, both ACh levels and contextual learning exhibited sex differences after puberty. These observations suggest that the postnatal changes in hippocampal ACh release cause sex differences in hippocampal learning function in postpubertal rats. Since bilateral microinjection of scopolamine into the dorsal hippocampus impairs contextual learning in both sexes of adult^39^,^40^ and juvenile rats^30^, the cholinergic activation of dorsal hippocampus seems to be necessary for contextual learning. As a cause of sex difference, we hypothesized sex-specific levels of circulating gonadal steroids after 6 weeks of age. Because serum testosterone in males and estradiol in females progressively increase after 6 weeks of age^41^, variation in the gonadal hormone environment may contribute to the sex differences in ACh release in the dorsal hippocampus and higher hippocampal learning performance in males. In male rats, gonadectomy decreases choline acetyltransferase immunoreactivity in medial septum and arm-choice accuracy during the acquisition of a working memory task in the radial arm maze^42^,^43^. In females, continuous treatment with estradiol in ovariectomized rats enhanced potassium-stimulated ACh release in the hippocampus and the acquisition of delayed matching to a position T-maze task^44^. We also reported that gonadectomy decreases hippocampal ACh levels in both sexes, while testosterone in orchidectomized rats or estrogen in ovariectomized rats restored the ACh release profile. These results suggest that corresponding sex hormones contribute to the maintenance of hippocampal ACh levels^21^.

Spontaneous behavior is an important factor to enhance hippocampal ACh levels. In fact, we have observed that the restriction of spontaneous activity attenuates both hippocampal ACh levels and spatial learning^19^,^45^,^46^, whereas intrahippocampal scopolamine infusion did not affect locomotor activity and baseline activity in the contextual learning paradigm^40^,^47^, suggesting the causal relationship between spontaneous behavior and hippocampal ACh levels. In the present study, juveniles exhibited much more spontaneous activity than adults when they showed equivalent ACh levels (Fig. 2). Moreover, voluntary running known to enhance the expression of BDNF mRNA and neurogenesis in the dentate gyrus improves both spatial^48^ and contextual learning^49^. These observations in rodents may be applicable in humans, because children in industrially or technologically advanced cities are more sedentary^50^. In addition to increasing risks of obesity and illness, the sedentary lifestyle also affects neurocognitive function and academic performance. A recent imaging study revealed that children who participate in high aerobic activity exhibited greater hippocampal volume and superior learning performance^50^, suggesting the importance of voluntary activity in education and public health. Finally, the released ACh may be associated with the development of contextual learning in juveniles, since long-term treatment of scopolamine after weaning impaired freezing without changing pain sensitivity (vocalization), emotional state (open field), and spontaneous activity (open field). The present findings, together with previous reports, lead to the hypothesis that juveniles require more spontaneous activity than adults to activate hippocampal functions. Our findings support the notion that every boy and girl requires sufficient spontaneous play in parks or nature to promote brain activity as well as physical activity.

## Methods

### Subjects

Male and female Wistar-Imamichi rats were obtained from Animal Reproduction Research Co. (Omiya, Japan). The rats were divided into six groups: 4-week-old males and females (juvenile), 6-week-old males and females in diestrus (pubertal), and 9- to 12-week-old males and females from diestrus 1 to diestrus 2 (adult). Developmental stages have been defined in previous reports^51^,^52^. Two to three rats from each group were housed in plastic cages (length 31 cm, width 47 cm, height 20 cm) at a constant temperature of 23 ± 1°C with a consistent light/dark cycle (lights on: 0500–1900 h). Food and water were available *ad libitum* during all experimental periods. In pubertal females, the vaginal opening was observed at postnatal 32 ± 1 days of age^53^. In pubertal males, testicular spermatozoa were consistently observed at postnatal 45 (but not 35) days of age^54^. Vaginal smears were obtained from female rats prior to the experiments, and the other groups of rats were handled for a short time each day^55^. All animal housing and surgical procedures were reviewed and approved by the Institutional Animal Care and Use Committee of the Animal Research Center, Yokohama City University and Yamaguchi University. All efforts were made to minimize the number of animals used and their suffering.

### Surgery

Under sodium pentobarbital anesthesia (31.5 mg/kg, i.p.), a stainless-steel guide cannula (outer diameter 0.51 mm; AG-4 or 8, Eicom Co., Kyoto, Japan) was implanted stereotaxically into the hippocampal CA1-3 subregion. In juvenile rats, the coordinates were 2.1–2.3 mm anterior to the bregma, 2.7–2.8 mm lateral to the midline, and 2.1 mm below the surface of the brain according to the atlas of Paxinos and Watson^56^. In pubertal rats, the coordinates were 2.6–2.9 mm anterior to the bregma, 2.9 mm lateral to the midline, and 2.1 mm below the surface of the brain. In adult rats, the coordinates were 3.1–3.5 mm anterior to the bregma, 3.0 mm lateral to the midline, and 2.1–2.2 mm below the surface of the brain. The anterior coordinate was adjusted based on sex and body weight^20^. After cannula implantation, a stylet was inserted into the guide until microdialysis. Each rat was individually housed in a cylindrical plastic cage (diameter 35 cm, height 45 cm), as described previously^57^,^58^.

### In vivo microdialysis

The *in vivo* microdialysis experiment was performed in an electromagnetic- and sound-shielded room (length 1.2 m, width 2.2 m, height 2.3 m)^18^. The day before the experiment, the stylet was replaced with a dialysis probe (AI-4-1 or 8-1, Eicom Co.) under light ether anesthesia. During the experiment, an artificial cerebrospinal fluid solution (147 mM NaCl, 4 mM KCl, 1.2 mM CaCl_2_, and 0.9 mM MgCl_2_) was infused through the dialysis probe with a 1.0-mm-long semi-permeable membrane at a rate of 1.2 μl/min using a microdialysis pump (CMA/102, Carnegie Medicin, Stockholm, Sweden) under unanesthetized, unrestrained conditions. Dialysates (24 μl) were automatically collected in an autoinjector (EAS-20, Eicom Co.) every 20 min for 24 h, and the same volume of ethylhomocholine solution (100 nM) was mixed in as the internal standard solution. This mixture was injected directly into a high performance liquid chromatography column every 20 min for the ACh assay.

**Figure 5:**
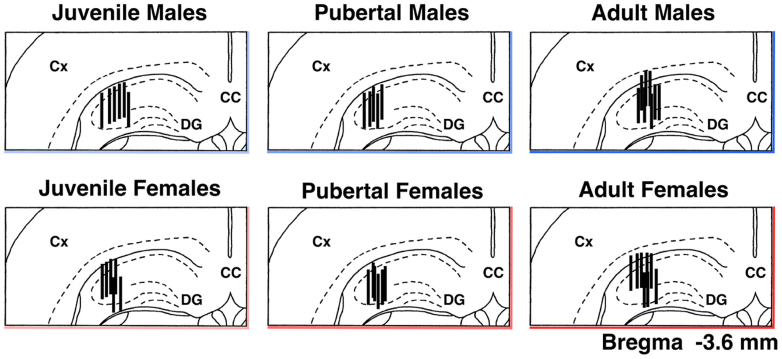
Location of the microdialysis probes within the dorsal hippocampus. Vertical lines represent dialysis membrane (length 1.0 mm). CC, corpus callosum; Cx, cortex; DG, dentate gyrus.

After sampling, the animals were deeply anesthetized with sodium pentobarbital and perfused with 10% formalin solution. Frozen coronal sections (50 μm thick) were sequentially cut from the forebrain using a microtome (MA-101, Yamato Koki Co., Miyagi, Japan). The location of the dialysis probe was microscopically verified in the frozen sections using a brain atlas^56^ (Fig. 5).

### Biochemical analysis of acetylcholine

The level of ACh in the dialysates was quantified using a combination of high performance liquid chromatography, enzyme assay, and electrochemical detection. The details of the ACh assay procedure were described previously^17^,^19^,^55^; however, no physostigmine was used in present study. Briefly, a solution of 0.1 mM Na_2_HPO_4_ (pH 8.5) containing 200 mg/l sodium 1-decanesulfonate (Aldrich Chemical Company Inc., Milwaukee, WI) was delivered as the mobile phase at a rate of 150 μl/min. After sample separation in a styrene polymer column (AC-GEL, Eicom Co.), ACh was converted to hydrogen peroxide using a post-column enzyme reactor (AC-ENZYMPAK, Eicom Co.) containing immobilized acetylcholinesterase and choline oxidase. The separation column and post-column reactor were maintained at 33°C. Hydrogen peroxide was detected with an electrochemical detector (HTEC-500, Eicom Co.). The lower limit of detection was 5–10 fmol/sample.

To calculate the recovery rate of each probe, *in vitro* experiments were also performed. The *in vitro* recovery rate was determined for individual probes and applied to the results from individual rats (mean ± S.E.M. 12.5 ± 1.8%, n = 39).

### Measurement of spontaneous locomotor activity

During the microdialysis study, the spontaneous locomotor activity of some rats was simultaneously monitored (n = 6 juvenile males, n = 6 juvenile females, n = 5 adult males, and n = 5 adult females). The housed cylindrical cages were placed on dielectric constant sensors with counters (ACTMONITOR II, Dia Medical System Co., Tokyo, Japan), and spontaneous locomotor activity was evaluated by measuring changes in the dielectric constant. Detected activity counts were recorded in a personal computer every 20 min for 24 h (VAIO PCG-Z1/P, Sony, Tokyo, Japan) using an interface unit (DAS-008, Neuroscience Inc., Tokyo, Japan)^45^,^59^. Activity counts were individually normalized by body weight using a previously described formula^60^.

### Fear conditioning test

Behavioral responses to contextual stimuli were checked in the sound-shielded room, as we reported previously^18^,^29^. A conditioning chamber (length 25 cm, width 31 cm, height 42 cm) was constructed of clear Plexiglass on the top, front, and back. The floor of the chamber was lined with 18 stainless steel bars (4 mm in diameter; 15 mm spacing) that formed a foot shock grid to deliver scrambled shocks produced by a stimulator (NS-SG01, Neuroscience, Tokyo, Japan).

In Figure 3, conditioning was performed 20 min after saline or scopolamine injection (2 mg/kg ip). In Figure 4, conditioning was performed one day after the final injection of saline or scopolamine (2 mg/kg sc). The rats were individually placed in the conditioning chamber and we started an audio-visual recording (Canon Inc, Tokyo, Japan). Rats were allowed to explore for 3 min. Then, a foot shock (2 sec duration, 0.8 mA), which served as the aversive unconditioned stimulus, was delivered three times. Vocalization was identified by frequency analysis using sound analyzing software (Sonic Visualiser v.1.8, developed by Queen Mary University of London, London, UK) as we reported previously^29^. Afterwards, the rats were allowed to recover for 30 sec in the conditioning chamber and returned to their home cage.

After the training session, the floor and interior of the conditioning chamber were cleaned with a 70% ethanol solution and dried. One hour later in Figure 3 or half hour later in Figure 4, the rats were again placed in the conditioning chamber and spontaneous behaviors were monitored for a 5-min period. To assess conditioning, we measured the time spent freezing per every 30 sec of the testing period. The time spent freezing (sec) in the chamber was considered the measure of contextual learning. Freezing behavior was defined as cessation of all but respiratory movement. This behavioral observation was performed on the second day of diestrus in adult females.

### Long-term scopolamine treatment

We obtained 3-week-old male rats and divided into 2 groups. Four rats from each group were housed in plastic cages (length 38 cm × width 23 cm × height 20 cm). Scopolamine hydrochloride (2.0 mg/kg) or corresponding volume of saline (30 to 100 μl) was subcutaneously injected twice daily (at 9:00 and 19:00) for a week. One day after the final injection, the juvenile rats were tested for open field test in the morning and fear conditioning test in the afternoon (Fig. 4).

### Open field test

We performed the open field test to evaluate emotional state and spontaneous locomotor activity. The field consisted of a circle arena (diameter 60 cm), with a gray floor divided into 2 areas (A center area of the apparatus (diameter 36 cm) and a peripheral area). After the audio-visual recording, we analyzed time spent in the center area and total traveled distance during 5 min.

### Statistical analysis

To evaluate sex differences, developmental changes, and time-dependent changes in hippocampal ACh release, extracellular ACh levels were analyzed using three-way analysis of variance (ANOVA) with repeated measures; the between-group factors were sex and age, and the within-group factor was time. Simple linear regression was used to evaluate the relationship between ACh levels and spontaneous locomotor activity. Pearson's correlation coefficient and the slope of the best-fit line were calculated for each individual rat. To evaluate sex differences and developmental changes in the correlation coefficient or the slope of the best-fit line, data were analyzed using two-way ANOVA; the between-group factors were sex and age. To evaluate sex differences, developmental changes, and the effects of shock exposure on the total freezing time, data were analyzed using three-way ANOVA; the between-group factors were sex, age, and conditioning. Significant interactions were further analyzed using *post-hoc* ANOVAs. Simple linear regression was used to evaluate the relationship between total freezing time and mean ACh levels. Pearson's correlation coefficient and the slope of the best-fit line were calculated for age groups. Unpaired *t*-test was used to evaluate long-term effect of scopolamine on behavioral tasks. Significance was set at *P* < 0.05.

## Author Contributions

K.T., S.Y. and D.M. performed the experiment. K.T. and D.M. designed the experiment and wrote the manuscript. F.K. and D.M. developed the experimental systems. All authors reviewed the manuscript.
